# Involving Patients in Hospital‐Based Health Technology Assessment of Innovative Medical Devices: Adapting to a Specific Local Context and Lessons Learned From the Assessment of an Ex Vivo Perfusion System of Human Donor Hearts

**DOI:** 10.1111/hex.70119

**Published:** 2024-12-11

**Authors:** Jamal Atfeh, Pascale Guerre, Alexandre Berkesse, Gwenaëlle Thual, Matteo Pozzi, Laure Huot

**Affiliations:** ^1^ Hospices Civils de Lyon, Pôle de Santé Publique, Service d'Evaluation Economique en Santé Lyon France; ^2^ Hospices Civils de Lyon, Unité d'évaluation des technologies de santé hospitalière Lyon France; ^3^ Université Claude Bernard Lyon 1, Research on Healthcare Performance RESHAPE Lyon France; ^4^ Université Claude Bernard Lyon 1, Health Systemic Process Lyon France; ^5^ Hospices Civils de Lyon, Direction qualité, usagers et santé populationnelle (DQUSP) Lyon France; ^6^ Institut du management, École des Hautes Études en Santé Publique (EHESP) Rennes France; ^7^ Hospices Civils de Lyon, Hôpital Louis Pradel, Service de Chirurgie Cardiaque Lyon France

**Keywords:** heart transplantation, hospital‐based health technology assessment, innovation, patient involvement

## Abstract

**Introduction:**

A demand from the cardiac surgery and heart transplantation department of a French (Lyon) university hospital to adopt an ex‐vivo perfusion system of human donor hearts was a chance to actively involve patients in our hospital‐based health technology assessment (HB‐HTA) process.

**Material and Methods:**

We selected an existing framework for patient involvement in HB‐HTA and involved patients at two stages of the HB‐HTA process: evaluation and dissemination. Firstly, we conducted a consultation‐oriented workshop to gather patient perspectives on the introduction of the technology in our hospital, based on their significant experience of healthcare. Secondly, we organized an information‐oriented workshop to communicate the HB‐HTA results to the patients consulted, after the decision had been taken.

**Results:**

We modified the framework for patient involvement to suit the local decision‐making context, the HB‐HTA methodology, and the type of technologies assessed in our institution. Patients perceived the ex‐vivo perfusion system as a promising technology to facilitate access to heart transplantation. They emphasized the importance of a tailored information provided to patients about the potential use of the technology in their healthcare trajectories, and suggested involvement of patients to facilitate its implementation in hospitals.

**Discussion:**

Modifying existing frameworks for patient involvement to fit specific local contexts should be encouraged, and has to address the need of timely information for decision‐makers and patient recruitment issues. Decision to incorporate patient perspectives and experiences should be made on a project‐by‐project basis, and focus on innovative medical devices with expected significant impact on patient quality of life. Effective and transparent communication and prospective feedbacks from HB‐HTA producers to patients are essential for a successful process.

**Patient or Public Contribution:**

Three patients with a lived experience of heart transplantation, or another transplant procedure, or more broadly procedures involving innovative medical devices (specialists) and two patients recruited for societal issues and legitimacy of a collective voice were involved (generalists).

## 1 Introduction

Health Technology Assessment (HTA) is a multidisciplinary field of research that emerged in the 1970s to study the medical, social, ethical and economic implications of the development, diffusion and use of health technologies [1, 2]. This process is widely used by national/regional health authorities regarding access of new health technologies to collectivity. However, hospitals often have to deal with demands for the introduction of innovative medical devices, which are available in the market but have not been evaluated at the national/regional level [3, 4]. Thus, local assessment of health technologies has been set up, taking into account the constraints and specificities of healthcare institutions, using the Hospital‐Based HTA (HB‐HTA) methodology [5]. Full HB‐HTA reports are generally based on a structured questionnaire in which the dimensions of interest are investigated [6]. Consideration of the social acceptability of health technologies is one of these key domains [7]. This involves integrating evidence of unique knowledge of the patient perspective and lived experience, which is of particular interest to improve the relevance of local assessments [6, 8, 9, 10].

In our institution, a dedicated team conducts HB‐HTA activities since 2008 to support local decision‐making for the adoption of innovative biomedical equipment. A demand from the cardiac surgery and heart transplantation department to adopt an ex‐vivo perfusion system for human heart transplantation required local assessment. Briefly, the ex‐vivo perfusion system – i.e., the TransMedics Organ Care System (OCS) Heart (TransMedics; Andover, MA) – is designed for the resuscitation, preservation, and assessment of human donor hearts [11]. Consisting of a console, a perfusion set, and solutions, it maintains hearts in a near‐physiological, normothermic and beating state to ensure optimal conditions for heart transplantation. Hearts are perfused with a warm donor blood‐based perfusate supplemented with nutrients and oxygen in a controlled and protected environment while the clinician can manage physiological parameters and system settings. This ex‐vivo perfusion system was proved non‐inferior to standard cold storage in terms of 30‐day patient and graft survival and significantly reduced the total ischemic time of the graft, which is a leading prognostic factor for primary graft survival [12, 13]. Its assessment properties could also enhance the utilization of extended criteria donor (ECD) hearts and therefore help in expanding the currently limited donor pool [14].

Given the target population and the potential public health benefit of this innovation, introducing a patient perspective in this assessment was considered relevant.

This request was therefore an opportunity to actively involve patients in our local assessment process for the first time. Based on this first experience, we were able to report patients' perceptions on the introduction of an ex‐vivo perfusion system of human donor hearts in our hospital and to share how an existing framework for patient involvement has been modified to suit our local context for the assessment of innovative medical devices [15]. Lessons learned from the process undertaken and perspectives for future assessments are discussed.

## 2 Methods

### 2.1 Theoretical Framework

We selected the framework for patient involvement in HTA at the local level proposed by Gagnon et al. [15]. Firstly, this framework explores which patients should be involved, distinguishing between ‘specialist’ patients – i.e., patients directly affected by the condition and/or treated with the technology evaluated – and ‘generalist’ patients – i.e., representatives of all current or potential service users – as conceptualized by Gauvin et al. [16]. Secondly, the framework by Gagnon et al. is based on three mechanisms of patient involvement commonly used in patient involvement literature: simple information (one‐way flow from HTA producers to patients); consultation (one‐way flow from patients to HTA producers); and participation (two‐way flow from both patients and HTA producers) [17, 18]. Finally, it highlights three different stages in the HB‐HTA process in which patients could be involved: selection of assessment topics (submitting requests and prioritizing topics); evaluation (drawing up the evaluation plan, collecting evidence with primary and secondary data, drafting the final report and formulating recommendations for implementation); and dissemination (developing information material and communicating HB‐HTA results).

### 2.2 Practical Application in a French University Hospital Setting

We analyzed the framework in relation to our local practices. For the local assessment of the ex‐vivo perfusion system of human donor hearts, we involved patients at two stages of the HB‐HTA process: evaluation and dissemination. We consulted patients on their perceptions of the introduction of the technology in our hospital at the evaluation stage and we informed them of the adopting decision at the dissemination stage. We involved in total five patients in this assessment: three specialist patients and two generalist patients according to Gagnon et al.'s framework.

#### 2.2.1 Patient Partnership Model at Lyon University Hospital

Our institution uses the pioneering Montreal model of partnership in care, which is often used as a framework for identifying the complementarity of knowledge and perspectives between health professionals and patients for enabling cooperation at the organizational level in care and research [19, 20]. An internal specialized unit (i.e., the *Partenariat et expérience patient en santé* – PEPS unit) has been dedicated to support health professionals in patient involvement in our institution. Patients involved in partnership projects at our institution are defined as follows. Patients as partners are volunteer patients identified by the PEPS unit or health professionals, who are involved because of their personal experience of living with the disease or their experience of health care, and the knowledge and skills they have developed through this experience [21]. Patient representatives have a legal status as they are appointed by a regional health authority on the recommendation of a recognized patient organisation with a 3‐year mandate, to speak on behalf of patients in bodies and working groups [21, 22]. These definitions slightly differed from the theoretical framework of Gagnon et al. but we could consider specialist patients as patient partners and generalist patients as patient representatives. This terminology will be used further.

#### 2.2.2 Ethical Considerations

Ethical approval was not required for this patient‐involvement project. Indeed, we did not collect personal health information but we did ask patients to help us evaluate the technology (as actors in the HB‐HTA process) based on what they learned from their significant healthcare experiences. Furthermore, patients involved were not current or future users of the health technology. However, patients involved in partnership projects at our institution systematically sign a partnership charter. This charter is designed to reflect the ethical conduct of partnership projects and the ethical management of patient contributions. It also ensures that the patients are clear about what their involvement means when they agree to participate. The patients involved in this project have therefore signed the charter.

#### 2.2.3 Recruitment and Selection of Patients

We solicited the physicians responsible for heart transplantation in the hospital to identify patients capable of mobilizing their own experience, and specialized patient organisations (gathering individual experiences of transplantation). In addition, patients already engaged in other partnership projects were contacted to evaluate if their healthcare experiences could be relevant to contribute. We recruited three specialist patients with lived experience of heart transplantation, or another transplant procedure, or a surgical procedure involving innovative medical devices. We also recruited two generalist patients for societal issues and the legitimacy of the collective voice.

#### 2.2.4 Consultation‐Oriented Workshop (Evaluation Stage)

We conducted the evaluation through an in‐person consultation‐oriented workshop. Members of the HB‐HTA team and the surgeon requesting the health technology were present during the workshop. Two members (hospital's adjunct director of quality of care and a methodological expert on patient involvement) of the PEPS unit facilitated the workshop, which was conducted as a discussion group, and gathered the patients for this face‐to‐face collective exercise [23].

After a preliminary introduction and preparation of the patients by the PEPS unit during the recruitment process, we explained the aim and expected results of the HB‐HTA process in details to all participants during the workshop, to provide them with all the necessary context. Special emphasis was given to its multidisciplinary nature (health professionals, HTA producers, patients, hospital managers) and on the dimensions explored (clinical, economic, organizational, patients' perceptions, strategic). We also presented the tool used to report these dimensions (i.e., the template developed by the AdHopHTA project [6]). It is structured as a series of 28 questions, one of which being dedicated to gather information on patients' perceptions (21 – Patients' perceptions: What is the patients' experience of the proposal/technology and its implications?) [6].

We also gave patients a description of the innovative medical device being evaluated, a summary of the existing literature on its intended use, and the expected local organizational and economic impacts on the institution. In this way, patients were able to properly express their perceptions by being aware of all the elements gathered by the experts in each field.

The workshop discussion began with the simple question: ‘What is your perception of the technology?’. Key domains then addressed by other questions during the workshop included (1) challenges in evaluating patient perspective (sharing insights on evaluation of the innovative medical device from the patient point of view and healthcare experiences); (2) expectations and concerns regarding the technology (discussion on expectations regarding the technology, and concerns or potential risks); (3) recommendations for implementation (guidance for a successful implementation of the technology within the hospital setting including the evaluation of the relevance of patient involvement during this specific phase).

At the end of the workshop, one of the facilitators assigned to take detailed notes during the workshop (hospital's adjunct director of quality of care) prepared a comprehensive summary capturing the key discussions, insights, and recommendations (no specific framework was used to structure the data). This summary was emailed to the participants for any necessary adjustments. The final version has been approved by all workshop participants, before being incorporated into the HB‐HTA report.

#### 2.2.5 Information‐Oriented Workshop (Dissemination Stage)

For effective communication and dissemination of the HB‐HTA results to patients who participated to the first workshop, we organized a second one to share the adopted decision and to allow for further discussions, if needed. The workshop was conducted remotely. Members of the HB‐HTA team and one member of the PEPS unit were present but the surgeon was unable to attend. A member of the HB‐HTA team shared the adopted decision and the member of the PEPS unit facilitated the workshop to outline any unresolved issues and get feedback on the global experience of involvement. Questions could be referred to the surgeon if needed.

## 3 Results

### 3.1 Analysis and Modification of the Gagnon et al. Framework to Fit the Local HB‐HTA Context

We modified the original framework to take into account the local decision‐making context, our HB‐HTA methodology, and the type of technologies (innovative medical devices) assessed in our institution.

The Gagnon et al. framework could not be fully implemented, firstly due to the top‐down process for the selection of assessment topics in our institution. Indeed, the hospital's governance directly commissions the HB‐HTA team with conducting its technology assessment requests and drafting the HB‐HTA reports. Therefore, getting suggestions from patients on assessment needs is not feasible and it is not possible to seek patient input on prioritization of topics given the current rate of assessments. Involving patients to draw up the evaluation plan (refinement of the question, dimensions to be evaluated, etc.) is not currently achievable considering our HB‐HTA process. Indeed, our reports are systematically drafted using the AdHopHTA questionnaire as the underlying framework for the dimensions that need to be evaluated to support local decision‐making, and to ensure the reproducibility of our assessments [6]. Furthermore, for the evaluation of innovative medical devices, patient‐related data is typically scarce in the literature due to low diffusion levels of technologies. Therefore, scheduling consultation‐oriented workshops to collect patients' views and perceptions on an innovative technology before deciding its adoption in hospitals is essential. The final report and recommendations are drafted by the HB‐HTA team based on inputs from various contributors within the institution, including the PEPS unit for patient dimension. Therefore, patients are indirectly involved in this phase of the HB‐HTA process, by gathering their experience. Patients can be involved in the development and dissemination of information material after adopting decisions in our institution. Information‐oriented workshops are feasible and essential for effective communication and dissemination of the HB‐HTA results to patients.

A summary of how the Gagnon et al. framework was modified to fit the local HB‐HTA context is given in Table 1.

**Table 1:** Existing framework for patient involvement modified to fit the local HB‐HTA context at Lyon University Hospital.

Theoretical framework	Practical application
**Selection of topics**	
Submitting requests	Top‐down process for selection of assessment topics (from hospital governance to the HB‐HTA team)
Prioritizing topics	Not applicable given the current rate of assessments
**Evaluation**	
Drawing up an evaluation plan	Evaluation plan based on AdHopHTA's mini‐HTA questionnaire
Collecting evidence (literature)	Patient‐related data is typically scarce in the literature for innovative medical devices
Collecting new data or contextualization	Patient involvement: Consultation‐oriented workshop
Final report and recommendations	Inputs from various contributors within the institution (PEPS unit for patient dimension)
**Dissemination**	
Development of material	Secondly after the adopting decision
Communication and dissemination of results	Patient involvement: Information‐oriented workshop

Abbreviations: HB‐HTA = hospital‐based health technology assessment, PEPS = Partenariat et expérience patient en santé.

*Source:* Modified from Gagnon et al. [15]

### 3.2 Consultation‐Oriented Workshop Findings

The workshop was held on January 18, 2022. Patients participating in the workshop unanimously acknowledged that they could not provide an opinion on the technical and medical aspects of the ex‐vivo perfusion system, and recognized challenging to assess its impact given the scarcity of currently available evidence in the literature. They expected a minor impact of the technology on patients' quality of life given that the device is not visible to patients and does not affect their daily life before or after transplantation. They also highlighted that it would make no difference to them which technology was involved, as long as they were adequately treated. The fact that the device was already used in other French institutions reassured the patients. Patients highlighted the potential benefits of the metabolic evaluation of the heart before transplantation, which could increase transplantation rates through wider use of available grafts. They found that these evaluative properties are very promising and could bring hope for severe patients awaiting heart transplantation. Concerns that some patients may experience stress and anxiety knowing that there is a risk that a graft will be declined for transplantation after evaluation by the device was debated, as this situation can arise during any transplantation, apart from the use of the device.

One of the main questions raised was whether patients should be informed about the potential use of an OCS™ Heart to perform their transplantation. Discussions emphasized that the psychological state of the patient could have an impact on their health outcomes and that the message should therefore be adapted to each patient (rather than a systematic discourse). For example, if the use of the ex‐vivo perfusion system is required for a complex heart transplantation (i.e. patient on long‐term mechanical circulatory support or with multiple previous sternotomies) or an ECD graft, this may raise concerns. Even if patients may not express a specific interest, the choice to be informed should be explicitly offered throughout the process. It may therefore be relevant for health professionals to inform the patient about the potential use of such technology during the initial pre‐transplantation consultation. Timing should also be carefully considered, as discussing it just before the transplantation procedure could affect the patient's emotional state.

All participants agreed on the importance of involving specialist patients to facilitate the technology implementation. They could indeed contribute to the development of appropriate information resources and provide support throughout the patient's hospital stay. Adding a mention of the use of the device in the surgical report was also suggested. Finally, patients reported that communicating about the acquisition of the device in an institutional and general manner could have a positive impact on the patient experience. Key findings of the consultation‐oriented workshop are summarized in Table 2.

**Table 2:** Key findings of the consultation‐oriented workshop.

**Challenges in evaluating patient perspective**
Challenging evaluation due to the scarcity of available evidence on the technology
Difficulties to provide opinions on technical and medical aspects
**Expectations regarding the technology**
Minor impact on patient's quality of life before or after transplantation
Great promise for patients awaiting heart transplantation especially given the graft evaluation properties, which could help expanding the donor pool
**Concerns regarding the technology**
Potential psychological distress and anxiety due to the utilization of the device for a complex heart transplantation (patient on long‐term mechanical circulatory support or with multiple previous sternotomies) or an ECD graft, or arising from possible graft rejection after evaluation with the ex‐vivo perfusion system
**Recommendations for implementation**
Information and discussion about the potential use of the technology with a discourse adapted to patient's psychological state during the initial pre‐transplant consultation
Mention the device in the surgical report if used
Involvement of patient partners to develop informational resources and provide support throughout the patient's hospital stay

Abbreviation: ECD = extended criteria donor.

Patients shared that they were pleasantly surprised to be consulted for the evaluation of this innovative device. They hoped that their input could contribute to a better‐informed decision regarding this technology, especially as it holds promise for heart transplant candidates and family caregivers. They also reported ease of integration into the group.

### 3.3 Dissemination and Feedback

A remote information‐oriented workshop was set up on January 24, 2023, once the local decision‐makers have reached the decision to adopt the device. All patients who participated in the consultation‐oriented workshop were present. This was highly appreciated and patients particularly valued that HB‐HTA producers took the time to share the decision orally. All participating patients reported a positive experience of their involvement in the HB‐HTA process. They did not point out any unanswered questions, unresolved issues or further concerns. Patients also expressed their desire to share the decision more broadly, which was allowed by the HB‐HTA team in compliance with certain confidentiality rules regarding sensitive information such as strategic issues for the institution.

## 4 Discussion

To the best of our knowledge, this is the first study reporting patients' perceptions on the introduction of an ex‐vivo perfusion system of human donor hearts in a hospital setting. Patients considered this device as a promising technology as it could improve preservation and evaluation of selected grafts and therefore help expanding the heart donor pool, which is currently limited. Consideration of emotional fluctuations and challenges faced in this situation by both patients and their families should be carefully taken into account by health professionals.

Unlike on national HTA, the literature on patient involvement in HB‐HTA is scarce and primarily documented through methodological works, mainly from Canada [10, 15, 24, 25]. Our study relied on the explicit modification of an existing framework for patient involvement in HTA at a local level for practical application, considering the HB‐HTA process of a specific institution. HB‐HTA inherently needs to be contextualized to properly support hospital managerial decisions [26, 27]. Therefore, we strongly believe that involving patients in HB‐HTA processes by modifying existing frameworks should be encouraged to take into account the unique and current decision‐making process of an institution. In ours, the direct application of the Gagnon et al. [15] framework faced challenges, in particular due to the top‐down demand for the assessment of innovative medical devices (the HB‐HTA team directly commissioned by hospital governance), and to the HB‐HTA methodology currently applied (reports systematically drafted following the template developed by the AdHopHTA project [6]). However, the decision‐making process of an institution is not set in stone and can evolve over time. For example, future perspectives could include establishing an advisory committee comprised of health professionals, hospital managers, members of the HB‐HTA unit and patients (participation as a mechanism of patient involvement) in a few years' time, with the aim of extending HB‐HTA activity to all healthcare technologies, whatever the source of demand within the institution.

Barriers for patient involvement in HB‐HTA mostly include acknowledged constraints in providing timely information for hospital decision‐makers [10]. Organizational timelines and patient involvement processes need to be formalized by implementing operational procedures to help address these time constraints. Anticipation of future demands of evaluation of health technologies is also crucial, particularly to address patient recruitment challenges. This is consistent with a prospective ambition to broaden the scope of patients involved in the HB‐HTA process of the institution by approaching patient organisations. The aim is to obtain a more representative opinion and to target the most relevant organisations according to the assessment being carried. Involving patients with experience of an HB‐HTA project as intermediaries with patient organisations to explain the aim and conditions of the process could contribute to their involvement. Indeed, the unique position of patient organisations allows for the inclusion of significantly diverse patient perspectives, but their involvement can face challenges due to their volunteer nature and their general lack of capacities due to financial constraints [28]. Patients who may have received the assessed innovative technology in other institutions could also be contacted. This would complement the assessment through patient involvement with qualitative research conducted in parallel. Indeed, one major challenge in evaluating innovative medical devices for their introduction in hospitals is to identify patients who could have received the technology (due to their low levels of diffusion). To continue evaluating the technology throughout its lifecycle, one solution might be to encourage qualitative research to be conducted once the technology is implemented in the institution, with patients treated with the technology evaluated. This research could be based on a focus group designed to collect patient perspectives on the technology. It could include the elaboration of a customized interview guide drafted in partnership with the patients involved in the technology assessment for its adoption, and should be based on existing resources and questionnaire templates proposed in the literature [29, 30]. The focus group could be audio recorded (with the consent of participants) to perform a qualitative verbatim analysis and complement the HB‐HTA with local qualitative data on patients' experiences with the technology.

Incorporating patient perspectives to the evaluation should be considered on a project‐by‐project basis [16]. The technologies addressed for assessment in our institution are innovative medical devices with strong hypotheses of clinical benefits, but substantial economic impacts for the institution. With regard to patient involvement in the HB‐HTA process, an emphasis should be made on technologies that have a great impact on patients' quality of life, require specific implementation in hospitals, or in which patients are actively involved [10].

Finally, in addition to the communication and dissemination of results of HB‐HTA, patients should be informed of contributions and arguments that influenced the adopting decision to fully achieve their involvement. Patients who participated to the assessment should also be given prospective feedback on whether the technology meets the expected benefits highlighted in the assessment, once it has been implemented in daily practice.

## 5 Conclusion

Involving patients for the local assessment of the ex‐vivo perfusion system of human donor hearts provided valuable insights into patient perspectives on this innovative technology. A theoretical patient involvement framework was modified to address our local decision‐making process. Perspectives for future assessments particularly include addressing patient recruitment challenges and constraints in providing timely information to hospital decision‐makers.

## Author Contributions

**Jamal Atfeh:** writing–original draft, writing–review and editing, conceptualization, methodology, investigation. **Pascale Guerre:** writing–review and editing, conceptualization, methodology, investigation, project administration, Validation. **Alexandre Berkesse:** writing–review and editing, methodology, investigation, validation. **Gwenaëlle Thual:** writing–review and editing, methodology, investigation, validation. **Matteo Pozzi:** writing–review and editing, supervision, investigation, validation. **Laure Huot:** writing–review and editing, conceptualization, methodology, supervision, investigation, project administration, validation.

## Conflicts of Interest

The authors declare no conflicts of interest.

## Data Availability

The authors have nothing to report.
